# The impact of increasing dose on overall survival in prostate cancer

**DOI:** 10.1186/s13014-015-0419-3

**Published:** 2015-05-21

**Authors:** Matthew D. Hall, Timothy E. Schultheiss, David D. Smith, Bertrand P. Tseng, Jeffrey Y. C. Wong

**Affiliations:** Department of Radiation Oncology, City of Hope National Medical Center, 1500 East Duarte Road, Duarte, CA 91010 USA; Division of Biostatistics, City of Hope National Medical Center, 1500 East Duarte Road, Duarte, CA 91010 USA; Duke University Medical Center, Durham, NC 27710 USA

**Keywords:** Radiation therapy, Prostate cancer, Dose, Dose response, Overall survival

## Abstract

**Purpose:**

To assess the impact of increasing dose on overall survival (OS) for prostate cancer patients.

**Methods:**

Treatment data were obtained on more than 20,000 patients in the National Oncology Data Alliance®, a proprietary database of merged tumor registries, who were treated for prostate cancer with definitive radiotherapy between 1995 and 2006. Eligible patients had complete data on total dose, T stage, use and timing of androgen deprivation therapy (ADT), and treatment start date (n = 20,028). Patients with prior malignancies were excluded.

**Results:**

On multivariate analysis, dose, T stage, grade, marital status, age, and neoadjuvant ADT were significant predictors of OS. Hazard ratios for OS declined monotonically with increasing dose, reaching 0.63 (95 % Confidence Interval 0.53–0.76) at ≥80 Gy. On subset analysis, neoadjuvant ADT significantly improved OS in high risk patients but was not significant in lower risk patients. The dose response was maintained across all risk groups. Medical comorbidities were balanced across all dose strata and sensitivity analysis demonstrated that other prognostic factors were unlikely to explain the observed dose response.

**Conclusions:**

This study suggests that increasing dose significantly improves OS in prostate cancer patients treated with radiotherapy.

**Electronic supplementary material:**

The online version of this article (doi:10.1186/s13014-015-0419-3) contains supplementary material, which is available to authorized users.

## Background

Dose-escalated radiotherapy provides superior freedom from clinical and biochemical failure in men treated for prostate cancer (PCa) [1–5] and is widely used in clinical practice [6]. Combined androgen deprivation therapy (ADT) and radiotherapy have demonstrated an OS advantage in intermediate and high risk patients in multiple randomized clinical trials (RCTs), albeit at doses ≤70 Gy [7–10]. Retrospective series have suggested that the benefit of dose escalation may be greater than that of ADT with conventional doses [11]. With a median follow-up of 7 years, Radiation Therapy Oncology Group (RTOG) 0126 showed significant improvements in the rates of biochemical failure and distant metastases with dose-escalated radiotherapy, but no OS benefit has been observed with higher doses to date [12].

The optimal radiotherapy dose must balance the risks and benefits of improved local control relative to treatment morbidity, but a treatment benefit in terms of improved OS is generally assumed [13, 14]. Given the potential toxicities of therapy and growing evidence that the treatment benefit in men with indolent PCa is limited [15, 16], it is important to analyze the outcomes of dose-escalated therapy in a population-based cohort to characterize this benefit where randomized data with adequate statistical power have been lacking. Therefore, the purpose of this study is to assess the impact on OS of increasing dose in PCa patients treated with definitive radiotherapy using a large database and to quantify the effects of prognostic factors.

## Methods

### Patient selection

Data on all men diagnosed with PCa from 1995 through 2006 were extracted from the National Oncology Data Alliance® (Elekta/IMPAC Medical Systems, Inc., Sunnyvale, CA and Stockholm, Sweden), a proprietary database of merged tumor registries. The registry is fully compliant with American College of Surgeons (ACOS) regulatory requirements. It captures newly diagnosed cancer cases at more than 150 hospitals in the United States. Data in the NODA registries are exactly the data sent to state tumor registries and to Surveillance, Epidemiology, and End Results (SEER), in regions that participate in SEER [17].

The study population included all patients treated with definitive radiotherapy with complete data on total dose, T stage, use and timing of ADT, and treatment start dates. Patients who underwent prostatectomy or brachytherapy, had distant disease, or had history of prior malignancy were excluded, leaving 20,028 evaluable patients. Patient characteristics included age, race, marital status, T stage, grade, total dose, ADT use, year of diagnosis, year of treatment, and medical comorbidities. In ACOS registries, Gleason scores are binned into four histologic grades [18]. PSA values were not available. This study was conducted with the approval of the City of Hope Institutional Review Board.

The primary endpoint was OS, measured from the start of treatment to the date of death from any cause. Participating institutions regularly update patient files with vital status. For patients reported alive at last follow-up, the Social Security death index (SSDI) was searched to extend follow-up duration. OS outcomes were updated through November 1, 2011, the last date that state death records were included in the Death Master File by the Social Security Administration as per Section 205r of the Social Security Act. Patients alive as of this date were censored.

### Statistical analysis

Statistical analysis was performed using SPSS v. 18.0 (SPSS Inc., Chicago) after patient-identifiers were removed from the data set. Multivariate proportional hazards analysis (MVA) was used to identify factors associated with OS [19]. The proportional hazards assumption was tested for a non-zero slope in the generalized linear model using scaled Schoenfeld residuals, and no substantial deviations were detected. Survival curves were plotted by Kaplan-Meier and compared using the log-rank test. *For survival analysis*, dose was considered as a categorical variable with *three dose bins* (68–71.99 Gy, 72–75.99 Gy, and ≥76 Gy). *For MVA*, the effect of dose was also analyzed as a categorical variable and was divided into *seven dose bins* with 68–69.99 Gy as the referent category. Correlation between significant prognostic variables and dose was examined by least squares regression and by plotting studentized residuals. MVA was performed with variates common to both the NODA and the Surveillance, Epidemiology, and End Results (SEER) database to confirm the validity of the data and the findings [17]. MVA yielded similar hazard ratios (HRs) and demonstrated congruence between the two databases (Additional file 1: Appendix 1). To address the potential differences between this retrospective study and RCTs, 5-year OS was calculated for our data and RCTs using high risk patients in both cases.

### Sensitivity analysis

The observed differences in OS between groups receiving different radiotherapy doses may reflect the effects of an unmeasured variable, like PSA, leading to a false association between dose and OS. For example, assigning patients to lower doses because they have higher PSAs and reduced life expectancy could mimic a dose response. Sensitivity analysis was performed to measure the potential influence of an unmeasured confounder on the hazard ratio (HR) estimate for OS. The association between dose and OS may be affected by the prevalences of the unmeasured confounder in the dose groups and its HR (*HR*_*confounder*_), which was assumed to be independent of dose. Only sampling bias for an unmeasured confounder with HR > 1 *and* higher prevalence in the lower dose group can mimic the dose response [20, 21]. Sensitivity analysis estimated the necessary prevalence to mimic the perceived dose response based on the presence of the confounder. Williams et al. showed that patients had significantly worse OS with pretreatment 40 ≥ PSA > 20 (HR = 1.32) and PSA > 40 (HR = 1.91), compared to patients with PSA < 10. [22]. Therefore, we modeled the effect of the putative confounder as a PSA imbalance using these published values.

Following the work of Lin et al., the degree of imbalance needed between dose groups to mimic the observed dose response is given by,

1 $$ {P}_{\mathrm{low}}=\frac{P_{high}}{H{R}_{dose}}-\frac{1}{H{R}_{dose}}\left(\frac{H{R}_{dose}-1}{H{R}_{confounder}-1}\right) $$

where *P*_*low*_ and *P*_*high*_ are the prevalences of the confounder in the low and high dose groups, respectively, and *HR*_*dose*_ is the observed HR for dose from the MVA (See Additional file 1: Appendix 2).

## Results

### Patients

Patient demographics and tumor characteristics are presented in Table 1. Median age was 71 years. ADT was administered in 40 % of patients as neoadjuvant or concurrent therapy with radiotherapy, defined as beginning within 6 months of the radiotherapy start date. The first notation for the use of intensity-modulated radiotherapy (IMRT) occurred in 2002. The prevalence of significant comorbidities was similar across all dose groups.

**Table 1:** Patient demographics

Dose		68–69.99 Gy	70–71.99 Gy	72–73.99 Gy	74–75.99 Gy	76–77.99 Gy	78–79.99 Gy	≥80 Gy
Number of patients		4,565	3,956	4,594	3,604	2,007	628	674
Age (Years)	Median	72	71	71	70	71	71	71
	Interquartile Range	[67, 76]	[66, 75]	[66, 75]	[65, 75]	[65, 75]	[66, 76]	[66, 75]
Race	White	85 %	81 %	84 %	82 %	85 %	82 %	86 %
	Black	12 %	16 %	14 %	15 %	12 %	16 %	12 %
	Asian/American Indian	1 %	1 %	1 %	1 %	1 %	1 %	1 %
	Unknown	2 %	2 %	1 %	2 %	2 %	1 %	1 %
Marital status	Single	18 %	17 %	20 %	20 %	22 %	21 %	23 %
	Married	74 %	74 %	72 %	67 %	69 %	68 %	72 %
	Unknown	8 %	9 %	8 %	13 %	9 %	11 %	5 %
T stage	T1	44 %	45 %	57 %	57 %	59 %	53 %	52 %
	T2	47 %	45 %	37 %	37 %	36 %	37 %	34 %
	T3	8 %	9 %	5 %	5 %	4 %	9 %	13 %
	T4	1 %	1 %	1 %	1 %	1 %	1 %	1 %
Grade	(1) Well Differentiated	11 %	8 %	3 %	2 %	2 %	1 %	1 %
	(2) Moderately Differentiated	57 %	58 %	59 %	55 %	45 %	41 %	42 %
	(3) Poorly Differentiated	19 %	23 %	33 %	40 %	51 %	54 %	55 %
	(4) Anaplastic	<1 %	<1 %	<1 %	<1 %	<1 %	1 %	1 %
	Unknown	13 %	11 %	5 %	3 %	2 %	3 %	1 %
High risk	T3-T4 or Grade 3–4	23 %	29 %	35 %	42 %	53 %	59 %	60 %
Androgen deprivation	No	68 %	59 %	53 %	54 %	54 %	50 %	59 %
Therapy	Yes	32 %	41 %	47 %	46 %	46 %	50 %	41 %
Year treated	1995–1997	54 %	31 %	5 %	1 %	1 %	1 %	<1 %
	1998–2000	32 %	40 %	15 %	5 %	3 %	3 %	<1 %
	2001–2003	11 %	19 %	37 %	30 %	20 %	14 %	16 %
	2004–2006	3 %	10 %	43 %	64 %	76 %	82 %	83 %
Medical	Cardiovascular Disease	13 %	19 %	16 %	15 %	21 %	25 %	18 %
Comorbidities	Diabetes mellitus	5 %	9 %	4 %	5 %	8 %	7 %	7 %
	Respiratory Disease	2 %	3 %	2 %	2 %	3 %	5 %	2 %

### Outcomes

**Fig. 1:**
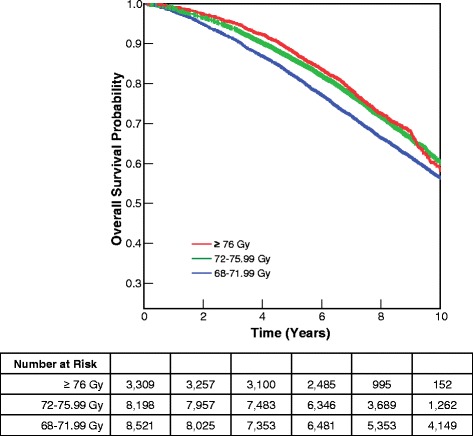
Overall survival (OS) by dose group. The Kaplan-Meier curves showed a clear and statistically significant association between higher radiotherapy dose and improved OS (p < 0.0001)

Median follow-up was 8.6 years for surviving patients. At the close-out date, 40 % of patients had died. The Kaplan-Meier curves showed a clear and statistically significant dose response relationship for OS (p < 0.0001) (Fig. 1). Median OS was 11.4 years (95 % Confidence Interval [CI] 11.1–11.6) for 68–71.99 Gy, 12.0 years (95 % CI 11.6–12.3) for 72–75.99 Gy, and 12.8 years (95 % CI 10.9–14.7) for ≥76 Gy.

**Fig. 2:**
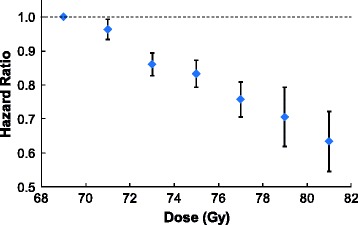
Hazard ratios for overall survival by dose group on multivariate analysis. Adjusting for the effect of other factors, hazard ratios for overall survival declined monotonically with increasing dose. Diamonds correspond to the observed hazard ratio for each dose group. The error bars indicate the standard error of the hazard ratio

Dose, T stage, grade, age, marital status, and neoadjuvant ADT were significant on MVA (Table 2). Race and treatment year were not significant. When dose was defined as a categorical variable with seven dose bins using 68–69.99 Gy as the referent category, the same variables were significant and no additional factors were significant. Adjusting for the effect of other prognostic factors, HRs for OS declined monotonically with increasing dose, reaching 0.63 (95 % CI 0.53–0.76) at ≥80 Gy, without evidence of a plateau (Fig. 2**)**. Even using the 74–76 Gy group (HR = 0.83, 95 % CI 0.77–0.90) as the referent category, treatment with ≥80 Gy maintained its significant OS advantage.

**Table 2:** Hazard ratios for overall survival

		p-value	HR	95 % CI
Dose (Gy)	68–69.99	<0.001	≡1.00	
	70–71.99	0.206	0.96	[0.91, 1.02]
	72–73.99	<0.001	0.86	[0.81, 0.92]
	74–75.99	<0.001	0.83	[0.77, 0.90]
	76–77.99	<0.001	0.76	[0.68, 0.84]
	78–79.99	<0.001	0.71	[0.59, 0.84]
	≥80	<0.001	0.63	[0.53, 0.76]
Androgen deprivation	No		--	
Therapy	Yes	0.009	0.94	[0.90, 0.98]
T stage	T1	<0.001	--	
	T2	<0.001	1.19	[1.14, 1.25]
	T3	<0.001	1.40	[1.29, 1.53]
	T4	<0.001	2.38	[1.90, 2.97]
Grade	(1) Well Differentiated	<0.001	--	
	(2) Moderately Differentiated	0.058	1.09	[1.00, 1.20]
	(3) Poorly Differentiated	<0.001	1.41	[1.28, 1.56]
	(4) Anaplastic	0.001	1.78	[1.25, 2.52]
Age	(Continuous)	<0.001	1.04	[1.04,1.05]
Marital status	Unmarried		--	
	Married	<0.001	0.80	[0.76, 0.85]

*HR* Hazard ratio, *CI* Confidence interval

On MVA, patients treated with higher doses also experienced a significant improvement in cause-specific survival (CSS). Using 68–71.99 Gy as the referent category, the HRs for CSS decreased sequentially to 0.62 (95 % CI 0.49–0.78) for 72–75.99 Gy and 0.26 (95 % CI 0.12–0.55) for ≥76 Gy.

As an exploratory analysis, patients were organized into high and low risk groups, approximating the National Comprehensive Cancer Network risk categories. Patients with either T3-T4 stage or Grade 3–4 histology were classified as high risk. In our analysis, T1 and T2, Grade 1 and T1, Grade 2 patients had comparable HRs for OS and were classified as low risk and T2 Grade 2 patients were categorized as intermediate. Dose retained significance on MVA for low, intermediate, and high risk patients, with comparable HRs for dose observed in all three subgroups. On subset analysis, ADT provided a significant OS benefit in high risk patients (HR 0.87, 95 % CI 0.81–0.94), but was not significant in lower risk patients on MVA (p > 0.5).

Dose was not correlated with age, race, marital status, T stage, grade, and neoadjuvant ADT. IMRT was first noted in patient records in 2002. When all patients treated in 2002 or later were excluded, dose remained statistically significant on MVA with small changes in the observed HRs. In the multivariate setting, there was no suggestion or trend of improved OS in later years (p > 0.7). Thus, the dose response does not appear to be an effect of treatment technique.

Treatment outcomes were examined with patients divided into three age subgroups (< 65, 65–74, and ≥75 years) to determine if the benefit associated with high dose treatment was greater in younger patients. HRs for OS were comparable in patients of all ages, adjusting for the effects of increasing age on mortality.

The association between dose and improved OS was evident in all subgroups examined, including stratification by ADT use. The observed OS advantage remained significant when patients who died within 6 months, 1, 2, 3, and 5 years of treatment were excluded, indicating that the benefit of high dose treatment was not related to an imbalance in the number of patients with abbreviated life expectancy consigned to receive lower doses. Reported medical comorbidities were balanced across all dose strata.

**Fig. 3:**
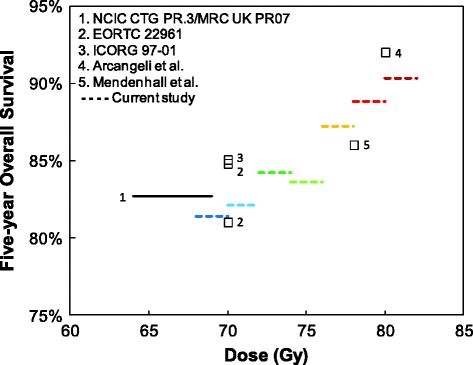
Five-year overall survival for randomized clinical trials in men with high risk prostate cancer treated with radiotherapy and androgen deprivation therapy. Also shown are data for high risk patients treated with androgen deprivation therapy in this study

Five-year OS estimates for our study and for RCTs enrolling high risk patients treated with external beam radiotherapy and ADT are shown in Fig. 3 [10, 23–26]. All patients in our study had a minimum 5-year follow-up, making 5-year OS a fair metric for comparison. Five-year OS was similar for RCTs and our cohort, suggesting not only that a very similar dose response was operative in both this study and the RCTs, but that the demographics and survival from non-PCa-related deaths were similar for all of these patient populations.

Sensitivity analysis was performed to assess the potential effects of an unmeasured confounder on the estimated HR for dose. The observed dose response could be simulated mathematically if patients in the high dose groups had lower pretreatment PSAs than patients in the lower dose groups. To determine the degree of PSA disparity needed to achieve the observed HR for OS, we assumed that all patients in the ≥80 Gy group had PSA < 10. We then calculated the prevalence of patients with elevated pretreatment PSA in the 68–70 Gy group that must be present to match the observed HR_*dose*_ = 0.63 (Table 2). To replicate the dose response, the 68–70 Gy group must have either 65 % of patients with PSA > 40 (HR = 1.91) and the remainder with PSA < 10 or 45 % with PSA > 40 and the remainder with 40 ≥ PSA > 20 (HR = 1.32) [22]. Thus, the median PSA must be approximately 40 in the 68–70 Gy group to account for the observed OS difference.

A disparity in the prevalence of a medical comorbidity, such as cardiovascular disease, could also have this effect, but the recorded prevalence of significant comorbidities was similar across all dose groups.

## Discussion

This observational study is the largest on PCa treated with known radiotherapy doses. On MVA, dose was a clear and statistically significant prognostic factor for OS without evidence of plateau up to 82 Gy. On subset analysis, ADT was protective in high risk patients but was not significant in low risk patients, whereas dose was significant in both low and high risk patients. The association between dose and improved OS was evident in all subgroups examined, including stratification by age and treatment with and without ADT. Although a retrospective analysis cannot account for all patient factors, the dose response was durable across a number of clinical scenarios and this analysis accounted for potential disparities in medical comorbidities. Sensitivity analysis also suggested that the presence of other uncoded variables, such as PSA, was unlikely to explain the observed dose response.

Several limitations in our study should be acknowledged. Although this study is the largest to address the impact of increasing dose on OS in patients with localized PCa treated with definitive radiotherapy, the data do not enable the opportunity to examine several important factors, including duration of ADT, use of pelvic nodal radiotherapy, and complications. Second, outcome measures were limited to OS and CSS, and data regarding local and distant failure cannot be extracted. Third, patient selection can influence the results in population-based observational series and must be considered when applying our results to clinical practice.

The completeness of the available data, however, helps to counter the possibility that the observed benefit of high dose radiotherapy is due solely to selection bias. First, the advantage of high dose remained when patients with shortened life expectancy (dying within 6 months to 5 years after treatment) were excluded. Second, the HRs for dose were consistent across all age groups, indicating that the benefit of high dose therapy was not biased by age discrepancies. Third, sensitivity analysis demonstrated that the observed dose response could be mimicked only by an unrealistic disparity in the prevalence of an unmeasured variable such as PSA that severely compromised survival. The agreement of this study with RCTs (Fig. 3**)** strongly imply that patients in this retrospective study were no more likely, at any dose, to have life-threatening comorbidities than patients in RCTs. Consequently, the accuracy of HR estimates may be affected by unmeasured variables, but reason dictates that these findings represent a true biological dose response.

RCTs provide precise measures and comparisons of treatment efficacy, have excellent internal validity, and are less prone to bias. Population-based observational studies cannot replace randomized data as the standard for outcomes research; however, it can provide evidence of the effectiveness of therapies in the general population and it can address questions that have not been adequately evaluated in RCTs [27]. The impact of bias can be minimized in observational studies by accounting for important covariates, including comorbidity, that are controlled in RCTs. In addition, the impact of unmeasured variables, such as PSA, can also be estimated using sensitivity analysis (as we did here). With careful planning, population-based observational studies can help to answer important clinical questions and fill the knowledge gaps left by existing randomized data.

Of note, PSA cannot be reliably extracted from US population-based databases before 2004, and changes in Gleason score measurements over time could result in upgrading of tumors diagnosed in later years [28].

Finally, increasing dose conferred a clear and statistically significant OS benefit, but the magnitude of benefit was modest. In this study, the median survival for patients treated with ≥76 Gy was 12.8 years compared to 11.4 years for 68–72 Gy. Men diagnosed with PCa have competing causes of mortality, including cardiovascular disease and second cancers [29, 30]. Appropriate patient selection is clearly an important consideration in the decision to treat that is beyond the scope of this study. Once this decision is made, however, our study suggests that treatment with higher dose and ADT in high risk patients confers a significant OS benefit. Only a prospective RCT can provide absolute measures of this advantage.

Five randomized dose escalation trials have shown superior freedom from failure at higher doses, but were underpowered to detect an OS difference [1–5]. In 393 patients receiving radiotherapy followed by proton boost to 70.2 vs. 79.2 GyE, Zietman et al. reported 5-year OS rates of 96 % and 97 %, respectively. Given the 96 % OS for the low dose group, no survival advantage could have been demonstrated in this study [1]. Kuban et al. reported no difference in 8-year OS in 303 patients treated with either 70 or 78 Gy. However, 73 % of patients were censored, salvage ADT was used in an unknown number of cases, and only 10 patients died of PCa [4]. In the largest dose escalation trial, Dearnaley et al. reported 10-year OS of 71 % in 843 patients treated with either 64 or 74 Gy. *All* patients received neoadjuvant and concurrent ADT, regardless of risk status [2].

RTOG 0126 demonstrated a significant reduction in biochemical failure and distant metastases with higher doses, but not an improvement in OS after a median follow-up of 7 years. Given the small number of CSS events to date, however, this finding is not unexpected and longer follow-up is required to credibly address this question, even in an adequately powered clinical trial [12].

In our study, increasing dose was associated with a significant OS benefit. The small incremental benefit of 1.4 years median survival, however, required thousands of patients to detect this difference at a level of statistical significance. A dose response can only be conclusively demonstrated in a clinical trial. To answer this question, two RCTs in the United States that were powered to detect an OS difference are maturing and ongoing. Our findings, however, suggest that although a dose response for OS exists, the magnitude of this advantage may compromise their ability to verify this outcome.

## Conclusions

In a large population-based cohort, our study suggests that OS is significantly improved in PCa patients in all risk groups treated with higher doses. The analysis also indicates that medical comorbidities, PSA, and other prognostic factors cannot credibly account for the observed dose response. We conclude that treatment with higher dose should be considered in all PCa patients receiving definitive radiotherapy provided that acceptable toxicity constraints can be achieved.

## Additional file

Additional file 1:
**Appendices.**
