# Supplementary material for: The impact of increasing dose on overall survival in prostate cancer
Source: Radiat Oncol. 2015 May 21;10:115. doi: 10.1186/s13014-015-0419-3 (PMC4448310; doi:10.1186/s13014-015-0419-3)
Supplement: Additional file 1: — Appendices. [file 13014_2015_419_MOESM1_ESM.pdf]

Appendix 1

Hazard ratios for overall survival on multivariate analysis for patients in the Surveillance, Epidemiology, and End Results (SEER) database and the National Oncology Data Alliance (NODA) from 1995-2006 with variates common to both datasets.

Table 3 Hazard Ratios for Overall Survival

		SEER			NODA		
		p-value	H.R.	95% C.I.	p-value	H.R.	95% C.I.
**T stage**	T1	<0.001	--		<0.001	--	
	T2	<0.001	1.15	[1.12, 1.18]	<0.001	1.21	[1.16, 1.27]
	T3	<0.001	1.49	[1.41, 1.57]	<0.001	1.42	[1.30, 1.54]
	T4	<0.001	2.42	[2.14, 2.74]	<0.001	2.41	[1.93, 3.01]
**Age**	(Continuous)	<0.001	1.05	[1.05, 1.06]	<0.001	1.05	[1.04, 1.05]
**Marital Status**	Unmarried		--			--	
	Married	<0.001	0.76	[0.74, 0.78]	<0.001	0.81	[0.77, 0.86]
**Grade**	(1) Well Differentiated	<0.001	--		<0.001	--	
	(2) Moderately Differentiated	0.516	1.02	[0.96, 1.09]	0.337	1.05	[0.95, 1.15]
	(3) Poorly Differentiated	<0.001	1.36	[1.27, 1.45]	<0.001	1.27	[1.16, 1.40]
	(4) Anaplastic	<0.001	1.88	[1.55, 2.28]	0.010	1.58	[1.12, 2.24]

Appendix 2

From Lin et al, "The proportional hazards regression (Cox, 1972) specifies that the hazard functions of T conditional on the sets of covariates (X, Z, U) and (X, Z) are, respectively,

$$\lambda(t|X,Z,U) = \lambda_O(t) \cdot \exp(\beta \cdot X + \gamma X \cdot U + \theta \cdot Z)$$

and

$$\lambda(t|X,Z,U) = \lambda_O^*(t) \cdot \exp(\beta^* \cdot X + \theta^* \cdot Z)$$

where $\lambda_O(\cdot)$ and $\lambda_O^*(\cdot)$ are arbitrary baseline hazard functions, and ($\beta, \gamma 0, \gamma 1, \theta$) and ($\beta^*, \theta^*$) are unknown regression parameters." X is the variate of interest, in this case dose, Z is a vector of other measured covariates, and U is an unmeasured confounder. β^* *and* θ^* represent the estimated parameter values determined in the absence of knowledge of the confounder. We assume that X and U take the value (0,1), where dose X=0 is the referent group, 1 is a nonreferent dose group, and U=0 and 1 represent the absence or presence of the confounder, respectively. We assume $\gamma 0 = \gamma 1 = \gamma$, meaning that the effect of the confounder is independent of dose.

Lin goes on to show that

$$\beta \approx \beta^* - ln\left(\frac{e^{\gamma} P_1 + (1 - P_1)}{e^{\gamma} P_0 + (1 - P_0)}\right)$$

where P_0 and P_1 are the prevalences of the confounder in the 0 and 1 dose groups respectively. Assuming equality, it can be shown that

$$\frac{HR_{dose}}{HR_{dose}^*} = \frac{HR_{confounder} P_0 + \left(1 - P_0\right)}{HR_{confounder} P_1 + \left(1 - P_1\right)}$$

If we assume that confounding accounts for all the dose response, then HR_{dose}=1. For P_1=P_{high} and P_0=P_{low}, we obtain equation (1) in the text.
